# Observational study of ocular surface squamous neoplasia: Risk factors, diagnosis, management and outcomes at a tertiary eye hospital in South Africa

**DOI:** 10.1371/journal.pone.0237453

**Published:** 2020-08-11

**Authors:** Roland Hӧllhumer, Susan Williams, Pamela Michelow

**Affiliations:** 1 Division of Ophthalmology, Department of Neurosciences, University of the Witwatersrand, Johannesburg, South Africa; 2 The Cornea Foundation, Johannesburg, South Africa; 3 Cytology Unit, National Health Laboratory Service and Department of Anatomical Pathology, Faculty of Health Sciences, University of the Witwatersrand, Johannesburg, South Africa; University of Pavia, ITALY

## Abstract

**Background:**

Ocular surface squamous neoplasia (OSSN) is the most common ocular surface tumour. Diagnosis and management have traditionally been by excision biopsy. Recently there has been success with the use of topical chemo or immunotherapy, which has resulted in a move from invasive diagnosis by histology to an array of non-invasive diagnostic tests.

**Methods:**

This observational study aims to describe the characteristics of patients with OSSN at St John Eye Hospital in Johannesburg, South Africa. Non-invasive diagnostic tests (impression cytology, anterior segment-OCT, methylene blue staining) will be compared to the gold standard, histology. Treatment success, recurrence and adverse events will be documented between three treatment options that include: surgical excision, topical 5-Fluorouracil (5FU) chemotherapy, and topical 5FU with retinoic acid therapy.

**Discussion:**

There is a trend to the use of less invasive diagnosis and management for OSSN. Minimally invasive diagnostic tests include cytology, anterior-segment OCT and methylene blue staining. The study will compare these to the gold standard histology, thereby providing evidence for their use in clinical practice. Interferon alpha 2b is commonly used as immunotherapy for OSSN. The cost of this medication is prohibitive to its adoption in a developing country. We therefore decided to use 5FU as the chemotherapeutic agent of choice in this study. The success, adverse events and recurrence rates with this agent may provide additional evidence for its use in the management of OSSN. Overall, if diagnosis and management can be implemented with good success in the outpatient environment, care can be improved for this condition in a developing country.

## Background

OSSN is the most common ocular surface tumour in sub-Saharan Africa (SSA) [1–3]. It includes a range of conjunctival neoplasia from pre-invasive to invasive lesions. Pre-invasive lesions include conjunctival intra-epithelial neoplasia (CIN, partial thickness epithelial dysplasia) and carcinoma in-situ (full thickness dysplasia). Conjunctival intra-epithelial neoplasia lies anterior to an intact basement membrane and is further divided into grade I-III, based on the degree of epithelial dysplasia. Lastly, the most severe form of OSSN is invasive squamous cell carcinoma (SCC), where dysplastic cells break through the conjunctival basement membrane [4, 5]. Untreated, OSSN can lead to blindness and even death. The reported incidence of OSSN is 0.03–1.9 per 100 000 persons/year in the United States and Australia, whereas the incidence in SSA is 1.6–3.4 per 100 000 persons/year [4, 6]. The difference between the two incidence rates has largely been attributed to the human immunodeficiency virus (HIV) pandemic in SSA [6].

Two main patterns of disease presentation have been identified; older male patients in temperate climates where HIV and HPV are not associated; and a younger female patient population in tropical climates where HIV and HPV are more prevalent [6]. SSA falls into the latter category with an estimated HIV infection rate of 13% in South Africa in 2018 [7].

### Risk factors

The leading risk factors for the development of OSSN are ultraviolet-B (UVB) radiation exposure and infection with the human papilloma virus (HPV) [4]. Other predisposing factors include: cigarette smoke exposure, vitamin A deficiency, ocular surface injury, chronic ocular inflammation (e.g. allergic conjunctivitis), exposure to petroleum chemicals, chronic viral infections (hepatitis B and C, HIV) and immunodeficiency [4, 8, 9].

The mutagenic effect of UVB is related to a combination of UVB induced DNA damage, primarily in the p53 tumour suppressor gene, and impaired DNA repair mechanisms [5, 10–13]. It has been found that spending more than 50% of time outdoors in the first 6 years of life and living within 30 degrees of the equator are UVB induced risk factors for OSSN [14].

HPV has been described as a risk factor for the development of OSSN [15]. HPV 16 and 18 have been identified as high-risk for the development of mucosal cancers, however their role in OSSN is still unclear [14, 16]. Cutaneous HPV types were first investigated by Ateenyi-Agaba [17] in 2004, who found cutaneous HPV types in 86% of SCC and 26% of controls. Studies following this have investigated both mucosal and cutaneous HPV types without consistent results [15, 17–38].

In HIV endemic countries, OSSN has been found to be the presenting feature of the HIV infection in 50–86% of patients [3, 6]. HIV increases the risk of OSSN by 8–19 fold, with the highest risk in the first 2 years of AIDS. HIV patients have an increase in the severity of OSSN, a greater likelihood of bilaterality, worse prognosis and a higher chance of recurrence [1, 3, 8, 39].

### Diagnosis

A diagnosis of OSSN is first suspected based on clinical appearance. The typical features on clinical examination are a vascularised interpalpebral conjunctival mass that may demonstrate leukoplakia, feeder vessels and a variable amount of pigmentation [4, 16, 40]. There may be extension of the lesion onto the adjacent cornea where it appears as a wavy superficial grey opacity [40, 41]. Morphologically it is classified as placoid, nodular or diffuse. The placoid type is further classified as gelatinous, papilliform or leukoplakic in appearance [5, 14, 41, 42]. In a large African study, it was found that using clinical features to make the diagnosis of OSSN only had a positive predicative value of 54% [43]. The gold standard for confirming the diagnosis is the histological analysis of a biopsy specimen. Several additional methods have been described for diagnosis and include: impression cytology (IC), anterior segment optical coherence tomography (AS-OCT), confocal microscopy, ultrasound biomicroscopy (UBM) and methylene blue stain [44]. There has been an increase in the use of these non-invasive techniques for diagnosis, as the management of OSSN has moved away from surgery, to topical chemo and immunotherapy [4, 16, 40, 45].

High resolution AS-OCT creates an in-vivo cross section of ocular tissue to a resolution of 5 μm. This allows the epithelium and stroma of the conjunctiva to be assessed. OSSN begins in the conjunctival epithelium and has three main features on AS-OCT: a thickened, hyperreflective epithelium; an abrupt transition in the appearance of the epithelium; and a clear plane between the lesion and the underlying stroma. A cut-off epithelial thickness of 142 μm has been described to distinguish OSSN from benign conjunctival lesions [45–47]. In a study by Shousha et al [48] all 19 patients with OSSN on histology had the classic features on AS-OCT.

IC is a minimally invasive technique whereby the superficial cells of the conjunctiva and cornea are collected by applying a nitrocellulose membrane to the ocular surface [2]. It has been shown to correlate with histological findings in 77–80% of cases and can be used to diagnose recurrent disease, monitor response to topical chemotherapy and distinguish clinically similar pathologies (limbal stem cell failure, pannus, OSSN) [2, 49].

Methylene blue is an acidophilic dye that has a selective affinity for nucleic acids and therefore has increased uptake by malignant cells, thereby causing staining. It has been shown to stain OSSN lesions with a sensitivity of 97% and specificity of 50% [44, 50].

### Management

OSSN management can be divided into two main groups, medical and surgical. There has been a move in recent years to medical therapy as this removes the risk associated with anaesthesia and surgery [2, 3, 51, 52].

Surgical management aims to remove the entire tumour in one piece and is indicated for tumours that occupy ≤4 clock hours of the limbus and have a basal diameter of less than 15mm [40]. The surgical approach follows the traditional “no touch” technique, which minimises seeding of the tumour during surgery, and recommends 3-4mm macroscopically clear margins. The main risks associated with surgery are limbal stem cell deficiency (LSCD), scarring, pyogenic granulomas, infection and damage to the sclera or retina from excessive cryotherapy. The limitation of surgery is that it only removes the macroscopically visible tumour. If the surgical margins are found to be involved on histology, adjuvant topical chemotherapy can be given to minimise recurrence [3, 4, 40, 52].

Medical management uses a single topical chemo or immunotherapy agent over a period of months. The agents that can be used include mitomycin-C (MMC), 5-fluorouracil (5FU) and interferon α2b (IFN). Each agent follows a specific regimen for a cycle of treatment. They may also be used for chemo-reduction to decrease the size of the tumour before surgery. The benefit of medical therapy is that it treats the entire ocular surface and negates the risks associated with surgery [3, 4, 16, 40]. Topical retinoic acid has been used in conjunction with topical IFN and has been found to have a synergistic effect by increasing its penetration [52–54].

Side effects of these agents vary, with the least side effects reported in the IFN group and the most reported in the MMC group. IFN is the best tolerated topical medication, however it is a very costly option and requires refrigeration. This makes 5FU an attractive option, as it has a low side effect profile, is cost effective and does not require refrigeration [55–63].

Patients who do not respond to surgery or chemotherapy can undergo plaque brachytherapy with strontium 90 or ruthenium 106 [64]. Failure to control the disease with a combination of the above treatment options will require an exenteration of the orbital tissue to prevent spread to adjacent sites.

### Outcomes

The primary outcomes of OSSN management are the resolution of tumours and minimising recurrence rates. Recurrence occurs mostly in the first 3–6 months after treatment. With surgical excision recurrence rates are 5–33%, even when surgical margins have been clear on histology, while recurrence rates with medical therapy range from 0–37% [16, 55, 62, 65]. Resolution rates among the medical treatment options (IFN, MMC, 5FU) have been shown to be similar, ranging from 90–96% [62]. In a study by Parrozzani et al [55] using 5FU the recurrence rate was 10% with a mean follow-up of 12 months and an average of 1.5 cycles of drops (1 cycle = 4 weeks of drops and 4 weeks drug holiday). Forty-eight percent of patients had mild side effects that resolved fully within 4 weeks of cessation of therapy [55]. The most common side effects with 5FU therapy are: epiphora, ocular discomfort, itching, photophobia, swelling, infection, superficial punctate keratopathy, filamentary keratitis, conjunctival redness and eyelid inflammation [52, 55, 62, 63]. The side effects can be mitigated with the use of artificial tears and topical corticosteroid drops [52, 64].

Risk factors for recurrence include HIV, nodular morphology and multi-focal lesions. A nodule of greater than 1.5mm thickness has been found to correlate with a poorer tumour response [8, 55, 62.

### OSSN research in Africa

Africa has been a focus for OSSN studies due to the higher prevalence of disease. Most of these studies have been conducted in east African countries including Uganda, Tanzania and Kenya. The high prevalence in these countries has largely been attributed to their proximity to the equator and the HIV pandemic [6].

There has been a paucity of studies in the South African context, with only two studies in reported literature [44, 66] Lecuona et al [66] reported their outcomes with the use of Strontium-90 brachytherapy and Steffen et al [44] determined the diagnostic accuracy of methylene blue stain for OSSN. There have been no comprehensive epidemiological, diagnostic, management and outcomes-based reports from a South Africa patient cohort.

South Africa is in the unique position to describe the outcomes of modern OSSN diagnosis and management techniques in a predominantly HIV patient group. We are also geographically able to offer unique insights into the risk factors of OSSN, as South Africa is situated at the border of the high risk UVB belt, but in a country of high HIV prevalence.

We therefore aim to describe the presentation, diagnosis, management and outcomes of patients with OSSN at a tertiary eye hospital in Johannesburg, South Africa.

## Study objectives

The objectives of this study are:

1. To describe the characteristics of patients with symptomatic conjunctival masses at St John Eye Hospital.
2. To compare non-invasive diagnostic methods (impression cytology, AS-OCT, methylene blue staining) with histology (gold standard) in the diagnosis of symptomatic conjunctival masses.
3. To evaluate the outcomes (success and recurrence rates) with OSSN treatment (medical and surgical) over a 2-year period.
4. To identify the characteristics associated with successful OSSN treatment and with OSSN recurrence over a 2-year period.
5. To describe the adverse events associated with surgical and medical treatment of OSSN.

## Methods and design

### Study design

This is an observational study of patients with symptomatic conjunctival masses at St John Eye Hospital (SJEH). SJEH is a tertiary eye hospital that provides ophthalmic services to the greater Soweto region, Johannesburg, South Africa. Ethics approval was obtained from the Human Research Ethics Committee at the University of the Witwatersrand (M190729). The study was registered with the Pan African Clinical Trials Registry on the 3^rd^ of December 2019 (PACTR201912900667480). Informed consent will be obtained from all participants.

### Study population and sample

All patients that present to SJEH from December 2019 will be considered for inclusion in the study. SJEH sees an average of 200 new cases of symptomatic conjunctival masses every year. Approximately 25% of these masses are benign pterygia with the remaining masses classified as OSSN on histology. We will recruit participants for 18 months. It is therefore expected that we will have a sample size of n = 300 (approximately 225 dysplastic and 75 benign) for objectives 1 and 2. Assuming a loss of 20%, the sample size for objective 3, 4 and 5 will be n = 180 participants. For objective 2 (diagnostic methods) a total sample size has been calculated at n = 173, for a sensitivity of 90%, to detect a difference of 10% with a 95% confidence interval.

Participants will be identified from new patients that present to SJEH with symptomatic conjunctival lesions. If these patients meet the inclusion and exclusion criteria, they will be invited to be part of the study and a formal consent will be obtained. If they do not meet these criteria or decline participation in the study, they will be offered the same management options.

Study data will be collected and managed using REDCap electronic data capture tools hosted at the University of the Witwatersrand. A separate excel spreadsheet with the study number and participant details will be kept on a password protected secure online database that will only be accessible to the principle investigator.

#### Inclusion criteria

Patients presenting with symptomatic conjunctival masses to SJEH that have:

1. Any suspicious features of OSSN on clinical examination
  - Leukoplakia
  - Feeder vessels
  - Pigmentation
2. Persistent symptoms despite topical medical therapy
  - Redness
  - Foreign body sensation
  - Blurred vision

#### Exclusion criteria

1. Age less than 18 years
2. Pregnant or breastfeeding
3. Previous surgery or topical chemotherapy in the presenting eye
4. Masses with invasion of adjacent structures: forniceal conjunctiva, palpebral conjunctiva, tarsal conjunctiva, lacrimal punctum and canaliculi, plica, caruncle, anterior or posterior eyelid lamellae, eyelid margin, and/or intraocular compartments.
5. Neurological conditions that prevent performing study investigations (AS-OCT, IC, methylene blue stain)
6. Heritable conditions that predispose to conjunctival tumours (Xeroderma pigmentosum and oculocutaneous albinism)
7. The presence of primary acquired melanosis

### Study outline

The study participants will have an electronic questionnaire completed and blood tests performed in order to describe their baseline characteristics. They will undergo histological diagnosis (incision or excision biopsy) as well as having three non-invasive diagnostics tests (impression cytology, AS-OCT, methylene blue staining). With confirmation of OSSN on histology, they will follow one of three treatment options that will be determined by the size of the lesion:

1. ≤4 limbal clock hours: surgical excision with adjuvant cryotherapy
2. >4 limbal clock hours and <1.5mm thick: topical 5FU chemotherapy
3. >4 limbal clock hours and ≥1.5mm thick: topical 5FU + retinoic acid chemotherapy

Participants undergoing surgical excision with positive margins on histology will receive one cycle of 5FU once the epithelium has healed. A cycle of 5FU 1% constitutes drop administration four times a day for a month with a drug holiday of one month. Retinoic acid 0.01% will be administered on alternate days for the duration of the 5FU cycles in participants with tumours ≥1.5mm thick. Participants will be assessed clinically for lymph node spread. If lymphadenopathy is identified a sentinel node biopsy will be performed. If lymph node spread is identified on biopsy a metastatic work-up will be performed. Participants diagnosed with OSSN will be followed-up for two years from clinical resolution of the lesion, to monitor for recurrence of the tumour. Recurrences will be managed with topical 5FU chemotherapy.

### Data collection

#### Baseline characteristics of the participants

Participant characteristics will be collected in the form of an electronic questionnaire and recorded on a REDCap database designed for the study. Clinical characteristics will be determined by a set of special investigations that include HIV, hepatitis B and C serology, vitamin A and C-reactive protein levels.

#### Comparison of diagnostic methods

All participants with symptomatic conjunctival lesions will undergo a histological diagnosis (excision or incision biopsy) as well as three non-invasive diagnostics tests (impression cytology, AS-OCT, methylene blue staining).

1. **Histology**
  If the mass is ≤4 clock hours the participant will be offered surgical excision and the tumour staged according to the AJCC 8^th^ edition. An informed consent will be taken for surgical excision of the mass. Surgery will be performed under local anaesthetic. Methylene blue will be instilled at the start of surgery to delineate the mass, which will be removed with a 4mm macroscopic tumour free margin using the classic no-touch technique with double cryotherapy if the mass has features suspicious of OSSN (leukoplakia, feeder vessels, pigmentation) If scleral invasion is found at surgery a lamellar sclerectomy will be performed with 2mm radial margins [40]. Masses larger than 4 clock hours have the risk of limbal stem cell failure after surgery. These participants will be offered an incision biopsy to confirm the histological diagnosis.
2. **Impression cytology**
  This will be performed before the start of surgery to minimise any participant discomfort. Local anaesthetic is used to anaesthetise the eye. Two nitrocellulose membranes will be applied sequentially to the surface of the mass for 10 seconds and placed in transport media.
3. **Anterior segment OCT**
  An AS-OCT will be performed of the mass to identify the following features:
  - Thickened, hyper-reflective epithelium
  - Abrupt transition from normal to abnormal epithelium
  - Plane between the lesion and underlying stroma
  The presence of two of these factors will used as indicative of OSSN.
4. **Methylene blue stain**
  A topical anaesthetic drop will be used, followed by a drop of methylene blue 1%. The eyelids will be closed for 30 seconds followed by a rinse with sterile water and an anterior segment photo taken [44]. This photo will be uploaded to the REDCap database. Stain uptake that is diffuse or focal will be regarded as a positive stain.

*Treatment success and recurrence*. Participants that are diagnosed with OSSN on histology will be managed according the size of the lesion:

1. ≤4 limbal clock hours: surgical excision with adjuvant cryotherapy
2. >4 limbal clock hours and <1.5mm thick: topical 5FU chemotherapy
3. >4 limbal clock hours and ≥1.5mm thick: topical 5FU + retinoic acid chemotherapy

Participants that receive 5FU, will continue with cycles until resolution and then have one more cycle. Participants will be considered to have successful treatment when the lesion is resolved clinically (macroscopically resolved mass) and on AS-OCT (normal epithelial profile). Participants that received topical 5FU will have a repeat impression cytology 6 months after the last cycle was completed. After clinical resolution (excision biopsy date or last cycle of 5FU), participants will be monitored for a recurrence for 24 months with follow-up visits at 3, 6, 9, 12 and 24 months.

Recurrence is diagnosed clinically and on AS-OCT. AS-OCT will be compared to the scan that was performed after treatment success. Changes in the epithelial profile together with the typical clinical appearance signify a recurrence. All recurrences will be managed with topical 5FU cycles. Participants who fail 5FU therapy (partial or no response) will be offered plaque radiotherapy (ruthenium-106) and those who fail plaque radiotherapy will be offered an exenteration.

*Adverse events*. Participants will have surgery related complications (LSCD, scarring, pyogenic granulomas, infection and damage to the sclera or retina) documented in the REDCap database. Those who use topical 5FU and retinoic acid will have side effects (epiphora, ocular discomfort, itching, photophobia, swelling, infection, superficial punctate keratopathy, filamentary keratitis, conjunctival redness and eyelid inflammation) of the drops monitored and documented at each visit. These side effects will be mitigated by the concurrent use of a topical anti-inflammatory and lubricant.

## Data analysis

Descriptive statistics will be used to describe participant characteristics. For normally distributed continuous variables, mean, standard deviation and 95% confidence interval will be used. For continuous variables that are not normally distributed and ordinal variables, median and interquartile range was will be used. For categorical variables, number, percentage and 95% confidence interval will be used. Regression analysis will be used to examine the relationship between conjunctival masses and the participants baseline characteristics.

Chi square test will be used to compare the sensitivity, specificity, positive predicative value and negative predicative value of the non-invasive diagnostic tests (impression cytology, AS-OCT and methylene blue stain) to histology. A ROC curve will be used to determine the optimum cut-off value for epithelial thickness on AS-OCT.

Descriptive statistics with number, percentage and 95% confidence interval will be used for the success and recurrence rates. The Kaplan-Meier curve will be used to compare the success and recurrence of the three treatment arms (surgery, 5FU, 5FU and retinoic acid). COX proportional hazard ratio will be used for a multivariate regression to determine the association of participant characteristics on success of management and recurrence for all three management options. Descriptive statistics with number, percentage and 95% confidence interval will be used to describe the surgery related complications and side effects of topical treatment.

## Discussion

OSSN is the most common ocular tumour and there has been a gradual increase in prevalence in SSA with the HIV pandemic [6]. Despite this there is a paucity of data for the South African context. South Africa is in the unique position to provide insight on the demographic and associated factors for patients that are not in the high risk UVB belt but have a high prevalence of HIV.

Diagnosis and management have traditionally been by excision biopsy using a no touch technique and adjuvant cryotherapy [5]. There has been a move to the use of non-invasive diagnostic and management techniques [40]. The use of AS-OCT has predominated the non-invasive diagnostic methods but has limitations. Firstly, sophisticated oncology units have access to custom made AS-OCT units with a higher resolution than commercially available units [45]. Additionally, patients in the South African setting often present with advanced tumours where AS-OCT is less useful to implement due to severe leukoplakia or thick masses. Because of this we have decided to focus our diagnosis on cytology as this is minimally invasive, can easily be performed in the outpatient setting, is easily repeatable and allows for additional testing such as PCR.

Medical management of OSSN can be with the use of IFN, 5FU or MMC [5]. Although IFN is the least toxic to the ocular surface, it has a significant cost factor and requires refrigeration. MMC is the most toxic to the ocular surface and requires refrigeration. We therefore decided to use 5FU as our medical agent of choice, as it causes minimal side effects, is cheap and does not require refrigeration. Evidence has shown benefit of adding retinoic acid to medical therapy and so we have added this as an adjuvant therapy to tumours thicker than 1.5mm to increase the efficacy of 5FU [53].

We are looking forward to reporting the sensitivity and specificity of cytology and the efficacy of medical therapy. If this is shown to be non-inferior to excision biopsy it will make a strong case for outpatient management of this condition. This will reduce patient morbidity and decrease the demand on hospital resources.

## Supporting information

S1 Checklist TREND statement checklist. (PDF)

S1 File (PDF)

## Abbreviations

5FU: 5-fluorouracil
AIDS: acquired immunodeficiency syndrome
AS-OCT: anterior segment optical coherence tomography
CIN: conjunctival intraepithelial neoplasia
HIV: human immunodeficiency virus
HPV: human papilloma virus
IC: impression cytology
IFN: interferon α2b
LSCD: limbal stem cell deficiency
MMC: mitomycin-C
OCT: optical coherence tomography
OSSN: ocular surface squamous neoplasia
PCR: polymerase chain reaction
SCC: squamous cell carcinoma
SJEH: St John Eye Hospital
SSA: sub-Saharan Africa
UBM: ultrasound biomicroscopy
UVB: ultraviolet-B
