# Supplementary material for: Observational study of ocular surface squamous neoplasia: Risk factors, diagnosis, management and outcomes at a tertiary eye hospital in South Africa
Source: PLoS One. 2020 Aug 11;15(8):e0237453. doi: 10.1371/journal.pone.0237453 (PMC7418961; doi:10.1371/journal.pone.0237453)
Supplement: S1 File — (PDF) [file pone.0237453.s002.pdf]

Ocular Surface Squamous Neoplasia: Risk factors, diagnosis, management and outcomes at a Tertiary Eye Hospital in South Africa

University of the Witwatersrand

Faculty of Health Sciences

Dr R Höllhumer

Contents

1. Introduction ... 3
    1.1. Diagnosis ... 5
    1.2. Management ... 8
    1.3. Outcomes ... 9
    1.4. OSSN research in Africa ... 10
2. Study Aim ... 11
3. Study Objectives ... 11
4. Methodology ... 12
    4.1. Study Design ... 12
    4.2. Study population and sample ... 12
    4.3. Study Outline ... 14
    4.4. Data collection ... 14
5. Data Analysis ... 19
6. Ethics and Dissemination ... 20
7. Timing ... 21
8. Funding ... 22
9. Competing Interests ... 22
10. References ... 23
11. Appendix A: Studies with primary or adjuvant use of 5FU for OSSN ... 30
12. Appendix B: Studies of OSSN in the African context ... 32
13. Appendix C: Information Sheet and Consent Form ... 39
14. Appendix D: Study Participant Details ... 40
15. Appendix E: Data Collection Sheet ... 41
16. Appendix F: Data Analysis ... 42
17. Appendix G: Budget ... 45

1. Introduction

A conjunctival mass is an inclusive term for benign growths of the conjunctiva, such as pterygia, as well as ocular surface squamous neoplasia (OSSN). Pterygia are benign fibrovascular growths of the conjunctiva that grow over the surface of the cornea and cause redness, irritation and blurred vision.[1] It has been found that up to 10% of pterygia have features of OSSN on histology.[2] OSSN is the most common ocular surface tumour in sub-Saharan Africa (SSA).[3–5] It includes a range of conjunctival neoplasia from benign to pre-invasive and invasive lesions. Benign lesions consist of conjunctival papillomas while pre-invasive lesions include conjunctival intra-epithelial neoplasia (CIN, partial thickness epithelial dysplasia) and carcinoma in-situ (full thickness dysplasia). Conjunctival intra-epithelial neoplasia lies anterior to an intact basement membrane and is further divided into grade I-III, based on the degree of epithelial dysplasia. Lastly, the most severe form of OSSN is invasive squamous cell carcinoma (SCC), where the dysplastic cells break through the conjunctival basement membrane.[6,7] Untreated, OSSN can lead to blindness and even death. The reported incidence of OSSN is 0.03 – 1.9 per 100 000 persons/year in the United States and Australia, whereas the incidence in SSA is 1.6 – 3.4 per 100 000 persons/year.[6,8] The difference between the two incidence rates has largely been attributed to the human immunodeficiency virus (HIV) pandemic in SSA.[8]

Two main patterns of disease presentation have been identified; older male patients in temperate climates where HIV and HPV are not associated; and a younger female patient population in tropical climates where HIV and HPV are more prevalent.[8] SSA falls into the latter category with an estimated HIV infection rate of 13.1% in South Africa in 2018.[9]

The leading risk factors for the development of OSSN are ultraviolet-B (UVB) radiation exposure and infection with the human papilloma virus (HPV).[6] Other predisposing factors include: cigarette smoke exposure, vitamin A deficiency, ocular surface injury, chronic ocular

inflammation (e.g. allergic conjunctivitis), exposure to petroleum chemicals, chronic viral infections (hepatitis B and C, HIV) and immunodeficiency.[6,10,11]

The mutagenic effect of UVB is related to a combination of UVB induced DNA damage, primarily in the p53 tumour suppressor gene, and impaired DNA repair mechanisms. The p53 gene is responsible for regulating the cell cycle in the G1-phase. If there has been DNA damage, the p53 protein is responsible for inducing cell cycle arrest to allow for the repair of the DNA before entering the cell cycle again. If the DNA damage is irreparable, it induces apoptosis. In so doing, it removes mutated cells from the cell cycle and inhibits tumour growth.[7,12–15]

It has been found that spending more than 50% of time outdoors in the first 6 years of life and living within 30 degrees of the equator are UVB induced risk factors for OSSN.[16] Clinical practice shows that OSSN occurs more commonly at the nasal limbus, however, this is not uniformly described in the literature. Coroneo[17] has provided a possible explanation for this nasal predominance of conjunctival lesions. He described the focusing effect of the cornea for temporal incident light and proposed that the intensity of light from the temporal aspect is increased by a factor of 20 by the normal cornea, thereby enhancing the UVB effect of radiation.

HPV has been described as a risk factor for the development of OSSN and can be detected with immunohistochemistry, in situ hybridisation and PCR (in order of increasing sensitivity).[18] HPV 16 and 18 have been identified as high-risk in the development of mucosal cancers, however their role in OSSN is still unclear.[16,19] Cutaneous HPV types were first investigated by Ateenyi-Agaba[20] in 2004, who found cutaneous HPV types in 86% of SCC and 26% of controls. Studies following this have investigated both mucosal and cutaneous HPV types without consistent results.[18,20–41]

In HIV endemic countries, OSSN has been found to be the presenting feature of the HIV infection in 50 - 86% of patients.[5,8] HIV increases the risk of OSSN by 8-19 fold, with the highest risk in the first 2 years of AIDS. HIV patients have an increase in the severity of OSSN, a greater likelihood of bilaterality, worse prognosis and a higher chance of recurrence.[3,5,10,42]

Vitamin A is required for the normal health of mucosal membranes, such as the conjunctiva, and a deficiency thereof has been cited as a risk factor for the development of OSSN.[7] As a fat soluble vitamin it is stored in the liver and released to maintain constant serum levels.[1] It has been shown that vitamin A deficiency is more common in HIV infected individuals both in the acute phase response and not in the acute phase response.[43] An acute phase response has been shown to artificially decrease serum vitamin A levels in the presence of normal vitamin A levels in the liver.[43,44]

1.1. Diagnosis

A diagnosis of OSSN is first suspected based on clinical appearance. The typical features on clinical examination are a vascularised interpalpebral conjunctival mass that may demonstrate leukoplakia, feeder vessels and a variable amount of pigmentation.[6,19,45] There may be extension of the lesion onto the adjacent cornea where it appears as a wavy superficial grey opacity.[45,46] Morphologically it is classified as placoid, nodular or diffuse. The placoid type is further classified as gelatinous, papilliform or leukoplakic in appearance.[7,16,46,47,47] In a large African study, it was found that using clinical features to make the diagnosis of OSSN only had a positive predicative value of 54%.[48] The gold standard for confirming the diagnosis is the histological analysis of a biopsy specimen. Several additional methods have been described for the diagnosis of OSSN and include: impression cytology (IC), anterior segment optical coherence tomography (AS-OCT), confocal microscopy, ultrasound biomicroscopy (UBM) and methylene blue stain.[49] There has been an increase in the use of these non-invasive techniques for

diagnosis, as the management of OSSN has moved away from surgery, to topical chemo and immunotherapy.[6,19,45,50]

Histology is the gold standard for OSSN diagnosis, with tissue derived from an excision or incision biopsy. Excision biopsy is preferred when the tumour occupies ≤4 clock hours of the limbus and has a basal diameter of less than 15mm, whereas an incision biopsy is preferred for larger tumours.[45]

High resolution AS-OCT creates an in-vivo cross section of ocular tissue to a resolution of 5 µm. This allows the epithelium and stroma of the conjunctiva to be assessed. OSSN begins in the conjunctival epithelium and has three main features on AS-OCT: a thickened, hyperreflective epithelium; an abrupt transition in the appearance of the epithelium; and a clear plane between the lesion and the underlying stroma. A cut-off epithelial thickness of 142 µm has been described to distinguish OSSN from benign conjunctival lesions. Thick or leukoplakic lesions may cause masking of the underlying stroma and make it difficult to assess the plane between the epithelium and stroma.[50–52] In a study by Shousha et al[53] all 19 patients with OSSN on histology had the classic features on AS-OCT. AS-OCT can also be used to assess response to topical therapy, as the epithelium returns to normal with successful treatment. It is however unclear what the exact chronological correlation is between histological and AS-OCT resolution, with treatment.[50] AS-OCT can also be used successfully in patients with multiple anterior segment pathologies (limbal stem cell deficiency, pannus, scarring) to identify OSSN within the complex ocular surface.[52]

IC is a minimally invasive technique whereby the superficial cells of the conjunctiva and cornea are collected by applying a nitrocellulose membrane to the ocular surface.[4] It has been shown to correlate with histological findings in 77 - 80% of cases and can be used to diagnose recurrent disease, monitor response to topical chemotherapy and distinguish clinically similar pathologies (limbal stem cell failure, pannus, OSSN).[4,54] It has been found to have a lower yield

in patients with significant surface keratin, as this limits the number of cells taken up by the filter paper.[54] Nolan et al[55] described the cytopathology of OSSN in detail and Barros et al[56] described a predictive index score that differentiates SCC from pre-invasive OSSN lesions. IC has several advantages that include[4]:

1. Provides a source of well-preserved cells from the ocular surface.
2. It limits the potential complications of ocular surgery such as scarring and limbal stem cell failure.
3. Minimally invasive and easy to perform on an out-patient basis.
4. There are no side-effects or contra-indications that have been noted and it is safe to use in children.
5. It can safely be repeated many times to monitor treatment or recurrence.
6. The sample can be assessed using several different technologies including cytological staining, PCR, immunoblotting analysis and flow cytometry.

Specimens from impression cytology can be processed using conventional cytological techniques or liquid based cytology (LBC). Liquid based cytology was originally developed for cervical smears and is an alternative method of specimen storage and processing whereby the collected material, instead of being spread onto a glass slide, is placed into a vial containing preservative fluid. Once in the laboratory, automated or semi-automated processes randomise cells, eliminate background blood and inflammation, and deposit a single layer of cells onto a glass slide. The glass slide is then stained, cover slipped and examined under the microscope. LBC is increasingly being utilised for non-gynaecologic specimens such as fine needle aspiration biopsy from various sites including thyroid, salivary gland, breast, lung, lymph nodes, and body cavity fluids. Advantages of LBC include simpler collection, standardised preparation, good cellular preservation, smaller area to examine under the microscope and the easy application of ancillary techniques e.g. HPV testing on the residual

fluid in the vial. Disadvantages include higher cost, the inability to rapidly review material on-site for adequacy of sample, slight morphologic differences compared to conventional cytology and that only Papanicolaou (and not Romanowsky) stains can be applied to the sample.[57–59]

Methylene blue is an acidophilic dye that has a selective affinity for nucleic acids and therefore has increased uptake by malignant cells, thereby causing staining. It has been shown to stain OSSN lesions with a sensitivity of 97% and specificity of 50%.[49,60]

There are no studies that compare a combination of the non-invasive techniques to histological analysis. Liquid based cytology has also never been used for impression cytology in OSSN.

1.2. Management

OSSN management can be divided into two main groups, medical and surgical. There has been a move in recent years to medical therapy as this removes the risk associated with anaesthesia and surgery. [4,5,61,62]

Surgical management aims to remove the entire tumour in one piece and is indicated for tumours that occupy ≤4 clock hours of the limbus and have a basal diameter of less than 15mm.[45] The surgical approach follows the traditional “no touch” technique, which minimises seeding of the tumour during surgery, and recommends 3-4mm macroscopically clear margins. An alcohol epitheliectomy with 2 mm margins on the cornea is performed if there is corneal extension. Adhesion of the mass to the underlying sclera requires a partial thickness lamellar sclerectomy with 2mm margins. Double freeze-thaw cryotherapy is applied to the free conjunctival border and limbus to lyse remaining tumour cells and promote vascular occlusion. The main risks associated with surgery are limbal stem cell deficiency (LSCD), scarring, pyogenic granulomas, infection and damage to the sclera or retina from excessive cryotherapy. The limitation of surgery is that it only removes the macroscopically visible tumour. If the surgical

margins are found to be involved on histology, adjuvant topical chemotherapy can be given to minimise recurrence.[5,6,45,62]

Medical management uses a single topical chemo or immunotherapy agent over a period of months. The agents that can be used include mitomycin-C (MMC), 5-fluorouracil (5FU) and interferon α2b (IFN). Each agent follows a specific regimen for a cycle of treatment. They may also be used for chemo-reduction to decrease the size of the tumour before surgery. The benefit of medical therapy is that it treats the entire ocular surface and negates the risks associated with surgery.[5,6,19,45] Topical retinoic acid has been used in conjunction with topical IFN and has been found to have a synergistic effect by increasing penetration thereof.[62–64]

Side effects vary with these agents, with the least side effects reported in the IFN group and the most reported in the MMC group. IFN is the best tolerated topical medication, however it is a very costly option and requires refrigeration. This makes 5FU (appendix A) an attractive option, as it has a low side effect profile, is cost effective and does not require refrigeration.[65,65–73]

Patients who do not respond to surgery or chemotherapy can undergo plaque brachytherapy with strontium 90 or ruthenium 106.[74] Failure to control the disease with a combination of the above treatment options will require an exenteration of the orbital tissue to prevent spread to adjacent sites.

1.3. Outcomes

The primary outcomes of OSSN management are the resolution of the tumours and minimising recurrence rates. Recurrence occurs mostly in the first 3-6 months after treatment. With surgical excision recurrence rates are 5 - 33%, even when surgical margins have been clear on histology, while recurrence rates with medical therapy range from 0 – 37%.[19,65,72,75] Resolution rates between the medical treatment options (IFN, MMC, 5FU) have been shown to be similar, ranging from 90 – 96%.[72] In a study by Parrozzani et al[65] using 5FU the recurrence rate was 10%

with a mean follow-up of 12 months and an average of 1.5 cycles of drops (1 cycle = 4 weeks of drops and 4 weeks drug holiday). Forty-eight percent of patients had mild side effects that resolved fully within 4 weeks of cessation of therapy.[65] The most common side effects with 5FU therapy are: epiphora, ocular discomfort, itching, photophobia, swelling, infection, superficial punctate keratopathy, filamentary keratitis, conjunctival redness and eyelid inflammation.[62,65,72,73] The side effects can be mitigated with the use of artificial tears and topical corticosteroid drops.[62,74]

Risk factors for recurrence include HIV, nodular morphology and multi-focal lesions. A nodule of greater than 1.5mm thickness has been found to correlate with a poorer tumour response.[10,65,72]

1.4. OSSN research in Africa

Africa has been a focus for OSSN studies due to the higher prevalence of disease (appendix B). Most of these studies have been conducted in east African countries including Uganda, Tanzania and Kenya.[3,10,12,15,29–31,33,36–38,40,48,49,73,76–93] The high prevalence in these countries has largely been attributed to their proximity to the equator and the HIV pandemic.

There has been a paucity of studies in the South African context, with only two studies in reported literature.[49,89] Lecuona et al[89] reported their outcomes with the use of Strontium-90 brachytherapy and Steffen et al[49] determined the diagnostic accuracy of methylene blue stain for OSSN. There have been no comprehensive epidemiological, diagnostic, management and outcomes-based reports from a South Africa patient cohort.

South Africa is in the unique position to describe the outcomes of modern OSSN diagnosis and management techniques in a predominantly HIV patient group. We are also geographically able to offer unique insights into the risk factors of OSSN, as South Africa is situated at the border of the high risk UVB belt, but in a country of high HIV prevalence.

Our study is additionally unique in the following ways:

1. This is the first study to use liquid-based cytology in the diagnosis of OSSN
    a. This is less invasive than the current gold standard
2. This is the first study to compare the different non-invasive diagnostic techniques to histological diagnosis
3. This is the first study that uses 5FU in the management of large OSSN tumours in the South African setting
4. This is the first study to use retinoic as an adjunct to 5FU for OSSN

2. Study Aim

The aim of the study is to describe the presentation, diagnosis, management and outcomes of patients with symptomatic conjunctival masses at a tertiary eye hospital in Johannesburg, in a country with a high prevalence of HIV.

3. Study Objectives

1. To describe the characteristics of patients that are associated with OSSN at St John Eye Hospital
2. To compare non-invasive diagnostic methods (impression cytology, AS-OCT, methylene blue staining) with histology (gold standard) in the diagnosis with OSSN
3. To evaluate the outcomes (success and recurrence rates) with OSSN treatment (medical and surgical) over a 1-year period
4. To identify the characteristics associated with successful OSSN treatment and with OSSN recurrence over a 1-year period
5. To describe the adverse events associated with surgical and medical treatment of OSSN

4. Methodology

4.1. Study Design

This is an observational study of patients with symptomatic conjunctival masses at St John Eye Hospital (SJEH). SJEH is a tertiary eye hospital that provides ophthalmic services to the greater Soweto region, Johannesburg, South Africa.

4.2. Study population and sample

All patients that present to SJEH from October 2019 will be considered for inclusion in the study. SJEH sees an average of 200 new cases of symptomatic conjunctival masses every year. Approximately 25% of these masses are benign pterygia with the remaining masses classified as OSSN on histology. We will recruit participants for 18 months. It is therefore expected that we will have a sample size of n=300 (approximately 225 dysplastic and 75 benign) for objectives 1 and 2. Assuming a loss of 20%, the sample size for objective 3, 4 and 5 will be n=180 participants. For objective 2 (diagnostic methods) a total sample size has been calculated at n=173, for a sensitivity of 90%, to detect a difference of 10% with a 95% confidence interval.

Participants will be identified from new patients that present to SJEH with symptomatic conjunctival lesions. New patients are screened on the day of first presentation to SJEH. If these patients meet the inclusion and exclusion criteria, they will be invited to be part of the study and a formal consent will be obtained and stored electronically in a secure password protected REDCap database (appendix C). If they do not meet these criteria or decline participation in the study, they will be offered the same management options.

Participants will be given a study number in the REDCap database. A separate excel spreadsheet (appendix D) with the study number and participant details will be kept on a password protected secure online database that will only be accessible to the principle investigator.

Inclusion criteria

Patients presenting with symptomatic conjunctival masses to SJEH that have:

1. Suspicious features of OSSN on clinical examination
    a. Leukoplakia
    b. Feeder vessels
    c. Pigmentation
2. Persistent symptoms despite topical medical therapy
    a. Redness
    b. Foreign body sensation
    c. Blurred vision

Exclusion criteria

1. Age less than 18 years
2. Pregnant or breastfeeding
3. Previous surgery or topical chemotherapy in the presenting eye
4. Masses with invasion of adjacent structures: forniceal conjunctiva, palpebral conjunctiva, tarsal conjunctiva, lacrimal punctum and canaliculi, plica, caruncle, anterior or posterior eyelid lamellae, eyelid margin, and/or intraocular compartments.
5. Neurological conditions that prevent performing study investigations (AS-OCT, IC, methylene blue stain)
6. Heritable conditions that predispose to conjunctival tumours (Xeroderma pigmentosum and oculocutaneous albinism)
7. The presence of primary acquired melanosis

4.3. Study Outline

The study participants will have an electronic questionnaire completed and blood tests performed in order to describe their baseline characteristics. They will undergo histological diagnosis (incision or excision biopsy) as well as having three non-invasive diagnostics tests (impression cytology, AS-OCT, methylene blue staining). With confirmation of OSSN on histology, they will follow one of three treatment options that will be determined by the size of the lesion:

1. ≤4 limbal clock hours: surgical excision with adjuvant cryotherapy
2. >4 limbal clock hours and <1.5mm thick: topical 5FU chemotherapy
3. >4 limbal clock hours and ≥1.5mm thick: topical 5FU + retinoic acid chemotherapy

Participants diagnosed with OSSN will be followed-up for one year from clinical resolution of the lesion, to monitor for recurrence of the tumour. Recurrences will be managed with topical 5FU chemotherapy.

4.4. Data collection

4.4.1. Characteristics of the participants

Participant characteristics will be collected in the form of an electronic questionnaire and recorded on a REDCap database designed for the study (appendix E). Clinical characteristics will be determined by a set of special investigations that include:

1. HIV

a. If the participant is known with HIV and has recent CD4 and viral load count, these data will be entered from the NHLS labtrak system.

b. If the participant is known with HIV and does not have recent CD4 and viral load counts, these will be tested.

c. If the participant has not been tested for HIV or reports to have tested negative, voluntary testing and counselling will be offered.

d. If the HIV test is positive, post-test counselling will be done and a CD4 and viral load will be performed.

e. The participant will then be referred to the HIV clinic at Chris Hani Baragwanath Hospital for initiation of anti-retroviral therapy. This process is routinely performed for all patients presenting with a conjunctival mass, as the leading risk factor in our population is HIV infection.[8]

f. If the HIV test is negative, post-test counselling will be performed.

2. HBV and HCV serology

a. Chronic HBV and HCV infection are risk factors for OSSN.[6]

b. If these are positive, the participant will be referred to the infectious disease clinic at Chris Hani Baragwanath Hospital.

3. Vitamin A-levels

Serum vitamin A levels will be tested. Vitamin A deficiency is a modifiable risk factor for OSSN.[6,43]

4. C-reactive protein (CRP)

This will need to be performed to determine whether the participant is in an acute phase response, in order to interpret the vitamin A levels and their association with OSSN. An acute phase response gives an artificially low serum vitamin A level.[43,44] A CRP of >10mg/L will be considered elevated and indicative of an acute phase response.

5. **Anterior segment photos**

Photos will be taken of the lesion at 10x and 16x magnification. These photos will be uploaded to the REDCap database for secure storage. The images will be used to classify the conjunctival masses into morphological types: leukoplakic, papillomatous, nodular, gelatinous, diffuse and fibrovascular.

4.4.2. Comparison of diagnostic methods

All participants with symptomatic conjunctival lesions will undergo a histological diagnosis (excision or incision biopsy) as well as three non-invasive diagnostics tests (impression cytology, AS-OCT, methylene blue staining).

1. **Surgical excision biopsy**

If the mass is ≤4 clock hours the participant will be offered surgical excision. An informed consent will be taken for surgical excision of the mass. Surgery will be performed under local anaesthetic. Methylene blue will be instilled at the start of surgery to delineate the mass, which will be removed with a 4mm macroscopic tumour free margin using the classic no-touch technique. If the corneal epithelium is involved, an alcohol epitheliectomy will be performed and if the mass is adherent to the sclera, a superficial sclerectomy will be performed. Lastly, if the mass has features suspicious of OSSN (leukoplakia, feeder vessels, pigmentation), cryotherapy will be applied twice to the free bulbar conjunctival margin and limbus.[45] This is the routine surgery performed for conjunctival masses at SJEH.

Participants will be discharged the same day with topical anti-biotics, steroid drops and analgesia. The first post-operative review will be performed in the first week after surgery to ensure adequate healing and to rule out any surgery related complications. Once the eye has healed fully, review will be done at 1 month.

With this visit the histology, impression cytology and blood results will be reviewed, and the records updated on REDCap. If there are any abnormalities on blood investigations the participant will be referred to internal medicine at Chris Hani Baragwanath hospital for review. If the histology showed any dysplasia with resection margin involvement, 1 cycle of topical 5FU will be given. If the histology revealed a benign conjunctival mass, the participant will be released from the study once the eye has fully healed from surgery.

2. Surgical incision biopsy

Masses that are larger than 4 clock hours have the risk of limbal stem cell failure after surgery. These participants will be offered an incision biopsy to confirm the histological diagnosis.

Participants will be discharged the same day with topical anti-biotics, steroid drops and analgesia. The first post-operative review will be performed in the first week after surgery to ensure adequate healing and to rule out any surgery related complications. Once the eye has healed fully, review will be done at 1 month.

With this visit the histology, impression cytology and blood results will be reviewed, and the records updated on REDCap. If there are any abnormalities on blood investigations the participant will be referred to internal medicine at Chris Hani Baragwanath hospital for review.

3. Impression cytology

This will be performed before the start of surgery to minimise any participant discomfort. Local anaesthetic is used to anaesthetise the eye. Two nitrocellulose membranes will be applied sequentially to the surface of the mass and placed in transport media for liquid-based cytology. This removes the surface cell layer from the

tumour for cytological analysis. As this only removes the surface layer of cells, it has no effect on the histology result from surgery.

4. Anterior segment OCT

An AS-OCT will be performed of the mass to identify the following features:

i. Thickened, hyper-reflective epithelium
ii. Abrupt transition from normal to abnormal epithelium
iii. Plane between the lesion and underlying stroma

The presence of two of these factors will used as indicative of OSSN.

5. Methylene blue stain

A topical anaesthetic drop will be used, followed by a drop of methylene blue 1%. The eyelids will be closed for 30 seconds followed by a rinse with sterile water and an anterior segment photo taken.[49] This photo will be uploaded to the REDCap database. Stain uptake that is diffuse or focal will be regarded as a positive stain.

4.4.3. Treatment success and Recurrence

Participants that are diagnosed with OSSN on histology will be managed according the size of the lesion:

1. ≤4 limbal clock hours: surgical excision with adjuvant cryotherapy
2. >4 limbal clock hours and <1.5mm thick: topical 5FU chemotherapy
3. >4 limbal clock hours and ≥1.5mm thick: topical 5FU + retinoic acid chemotherapy

Participants that receive 5FU, will continue with cycles until resolution and then have one more cycle. Participants will be considered to have successful treatment when the lesion is resolved clinically and on AS-OCT. Participants that received topical 5FU will have a repeat impression cytology 6 months after the last cycle was completed. After clinical resolution (excision biopsy date or last

cycle of 5FU), participants will be monitored for a recurrence for 12 months with follow-up visits at 3, 6, 9 and 12 months. This is standard practice in OSSN management.

Recurrence is diagnosed clinically and on AS-OCT. AS-OCT will be compared to the scan that was performed after treatment success. Changes in the epithelial profile together with the typical clinical appearance signify a recurrence. All recurrences will be managed with topical 5FU cycles. Participants who fail 5FU therapy will be offered plaque radiotherapy and those who fail plaque radiotherapy will be offered an exenteration.

4.4.4. Adverse events

Participants will have surgery related complications (LSCD, scarring, pyogenic granulomas, infection and damage to the sclera or retina) documented in the REDCap database. Those who use topical 5FU and retinoic acid will have side effects (epiphora, ocular discomfort, itching, photophobia, swelling, infection, superficial punctate keratopathy, filamentary keratitis, conjunctival redness and eyelid inflammation) of the drops monitored and documented at each visit. These side effects will be mitigated by the concurrent use of a topical anti-inflammatory and lubricant.

5. Data Analysis

Descriptive statistics will be used to describe participant characteristics (appendix F). For normally distributed continuous variables, mean, standard deviation and 95% confidence interval will be used. For continuous variables that are not normally distributed and ordinal variables, median and interquartile range was will be used. For categorical variables, number, percentage and 95% confidence interval will be used. Regression analysis will be used to examine the relationship between conjunctival masses and the participants baseline characteristics.

Chi square test will be used to compare the sensitivity and specificity of the non-invasive diagnostic tests (impression cytology, AS-OCT and methylene blue stain) to histology. A ROC curve will be used to determine the optimum cut-off value for epithelial thickness on AS-OCT.

Descriptive statistics with number, percentage and 95% confidence interval will be used for the success and recurrence rates. The Kaplan-Meier curve will be used to compare the success and recurrence of the three treatment arms (surgery, 5FU, 5FU and retinoic acid). COX proportional hazard ratio will be used for a multivariate regression to determine the association of participant characteristics on success of management and recurrence for all three management options. Descriptive statistics with number, percentage and 95% confidence interval will be used to describe the surgery related complications and side effects of topical treatment.

6. Ethics and Dissemination

This proposal will be submitted to the Human Research Ethics Committee at the University of the Witwatersrand for clearance before commencing data collection.

Study participants will still receive the current gold standard of treatment i.e. surgical excision/topical chemotherapy and histologic diagnosis. If patients do not wish to participate in the study, their management will not be compromised in any way.

Patients will be allocated a study number. Data will be recorded, collated and stored electronically on a secure REDCap database that is password protected, with no patient identifiers. No information other than that specified on the data collection sheet will be collected. There will be loss of benefits to participants if they withdraw from the study.

This PhD will be completed by publication and presented at the annual congress of the Ophthalmology Society of South Africa. The themes for the publications will be:

1. Characteristics of patients presenting with OSSN at a Tertiary eye hospital in Johannesburg.
2. A comparison of histology and non-invasive diagnostic methods (impression cytology, AS-OCT, methylene blue staining) diagnostic methods for OSSN.
3. Evaluating the outcomes of OSSN treatment success and recurrence over a one-year period.
4. Factors associated with OSSN treatment success and recurrence.

This study will have no financial implications to the participants or St John Eye Hospital.

7. Timing

Data collection will commence after ethics approval has been granted. This is expected to commence from October 2019 until the sample size has been reached. Follow-up will continue for 1 year after resolution of the conjunctival lesions.

	**2019 Semester 1**	**2019 Semester 2**	**2020 Semester 1**	**2020 Semester 2**	**2021 Semester 1**	**2021 Semester 2**	**2022 Semester 1**	**2022 Semester 2**
**Literature Review**								
**Preparing Protocol**								
**Protocol Assessment**								
**Ethics Application**								
**Collecting Data**								
**Data Analysis**								
**Writing up papers**								

8. Funding

Funding for the methylene blue and filter paper used in this study will be from the principle researchers RINC fund at the University of the Witwatersrand. Transport medium for the specimens and filters required for the processing will be supplied by the NHLS. Retinoic acid 0.01% drops will be supplied from Fagron without a charge. This is a compounding pharmacy that makes up these drops. The remaining management is standard treatment protocol for this condition and will not require any additional funding. The filters for use in the laboratory will require funding that has been secured from the Carnegie Research Fund (appendix G).

9. Competing Interests

There are no conflicts of interest in this research.

10. References

1. Krachmer JH, Mannis MJ, Holland EJ, eds. *Surgery of the Cornea and Conjunctiva*. 3. ed. Maryland Heights, Miss.: Mosby Elsevier; 2011.

2. Hirst LW, Axelson R, Schwab I. Pterygium and Associated Ocular Surface Squamous Neoplasia. *Arch Ophthalmol*. 2009;127:31. doi:10.1001/archophthalmol.2008.531.

3. Steele KT, Steenhoff AP, Bisson GP, et al. Ocular surface squamous neoplasia among HIV-infected patients in Botswana. *S Afr Med J*. 2015;105:379. doi:10.7196/SAMJ.8254.

4. Barros J de N, Almeida SRA de, Lowen MS, et al. Impression cytology in the evaluation of ocular surface tumors: review article. *Arq Bras Oftalmol*. 2015;78:126-132. doi:10.5935/0004-2749.20150033.

5. Cicinelli MV, Marchese A, Bandello F, et al. Clinical Management of Ocular Surface Squamous Neoplasia: A Review of the Current Evidence. *Ophthalmol Ther*. 2018;7:247-262. doi:10.1007/s40123-018-0140-z.

6. Shields CL, Chien JL, Surakiatchanukul T, et al. Conjunctival Tumors:Review of Clinical Features, Risks, Biomarkers, and Outcomes—The 2017 J. Donald M. Gass Lecture. *Asia-Pac J Ophthalmol*. 2017;6:109-120. doi:10.22608/APO.201710.

7. Basti S, Macsai MS. Ocular Surface Squamous Neoplasia. 2003;22:18.

8. Gichuhi S, Sagoo MS, Weiss HA, et al. Epidemiology of ocular surface squamous neoplasia in Africa. *Trop Med Int Health*. 2013;18:1424-1443. doi:10.1111/tmi.12203.

9. Statistics South Africa. Mid-year population estimates: 2018. 2018:26.

10. Gichuhi S, Macharia E, Kabiru J, et al. Risk factors for ocular surface squamous neoplasia in Kenya: a case-control study. *Trop Med Int Health*. 2016;21:1522-1530. doi:10.1111/tmi.12792.

11. Napora C, Cohen EJ, Genvert GI, et al. Factors associated with conjunctival intraepithelial neoplasia: a case control study. *Ophthalmic Surg*. 1990;21:27-30.

12. Ateenyi-Agaba C. TP53 mutations in squamous-cell carcinomas of the conjunctiva: evidence for UV-induced mutagenesis. *Mutagenesis*. 2004;19:399-401. doi:10.1093/mutage/geh048.

13. Gichuhi S, Ohnuma S, Sagoo MS, et al. Pathophysiology of ocular surface squamous neoplasia. *Exp Eye Res*. 2014;129:172-182. doi:10.1016/j.exer.2014.10.015.

14. Tananuvat N, Lertprasertsuke N. Ocular Surface Squamous Neoplasia. *Intraepithelial Neoplasia*.:30.

15. Tornesello ML, Waddell KM, Duraturo ML, et al. TP53 codon 72 polymorphism and risk of conjunctival squamous cell carcinoma in Uganda. *Cancer Detect Prev*. 2005;29:501-508. doi:10.1016/j.cdp.2005.08.003.

16. Lee GA, Hirst LW. Ocular Surface Squamous Neoplasia. *Surv Ophthalmol*. 1995;39:429-450.

17. Coroneo M. Ultraviolet Radiation and the Anterior Eye: *Eye Contact Lens Sci Clin Pract*. 2011;37:214-224. doi:10.1097/ICL.0b013e318223394e.

18. Asadi-Amoli F, Heidari AB, Jahanzad I, et al. Detection of Human Papillomavirus in Squamous Cell Carcinoma of Conjunctiva by Nested PCR: A Case Control Study in Iran. :8.

19. Mittal R, Rath S, Vemuganti GK. Ocular surface squamous neoplasia – Review of etio-pathogenesis and an update on clinico-pathological diagnosis. *Saudi J Ophthalmol*. 2013;27:177-186. doi:10.1016/j.sjopt.2013.07.002.

20. Ateenyi-Agaba C, Weiderpass E, Smet A, et al. Epidermodysplasia verruciformis human papillomavirus types and carcinoma of the conjunctiva: a pilot study. *Br J Cancer*. 2004;90:1777-1779. doi:10.1038/sj.bjc.6601743.

21. McDonnell JM, Mayr AJ, Martin WJ. DNA of human papillomavirus type 16 in dysplastic and malignant lesions of the conjunctiva and cornea. *N Engl J Med*. 1989;320:1442-1446. doi:10.1056/NEJM198906013202202.

22. Lauer SA, Malter JS, Ralph Meier J. Human Papillomavirus Type 18 in Conjunctival Intraepithelial Neoplasia. *Am J Ophthalmol*. 1990;110:23-27. doi:10.1016/S0002-9394(14)76932-6.

23. Waddell K, Magyezi J, Bousarghin L, et al. Antibodies against human papillomavirus type 16 (HPV-16) and conjunctival squamous cell neoplasia in Uganda. *Br J Cancer*. 2003;88:2002-2003. doi:10.1038/sj.bjc.6600950.

24. Tabrizi SN, McCurrach FE, Drewe RH, et al. Human papillomavirus in corneal and conjunctival carcinoma. *Aust N Z J Ophthalmol*. 2007;25:211-215. doi:10.1111/j.1442-9071.1997.tb01394.x.

25. Palazzi MA, Erwenne CM, Villa LL. Detection of human papillomavirus in epithelial lesions of the conjunctiva. *Sao Paulo Med J*. 2000;118:125-130. doi:10.1590/S1516-31802000000500003.

26. Eng H-L, Lin T-M, Chen S-Y, et al. Failure to Detect Human Papillomavirus DNA in Malignant Epithelial Neoplasms of Conjunctiva by Polymerase Chain Reaction. *Am J Clin Pathol*. 2002;117:429-436. doi:10.1309/RVUP-QMU3-5X6W-3CQ1.

27. Scott IU, Karp CL, Nuovo GJ. Human papillomavirus 16 and 18 expression in conjunctival intraepithelial neoplasia. *Ophthalmology*. 2002;109:542-547. doi:10.1016/S0161-6420(01)00991-5.

28. Tulvatana W. Risk factors for conjunctival squamous cell neoplasia: a matched case-control study. *Br J Ophthalmol*. 2003;87:396-398. doi:10.1136/bjo.87.4.396.

29. Moubayed P, Mwakyoma H, Schneider DT. High Frequency of Human Papillomavirus 6/11, 16, and 18 Infections in Precancerous Lesions and Squamous Cell Carcinoma of the Conjunctiva in Subtropical Tanzania. *Am J Clin Pathol*. 2004;122:938-943. doi:10.1309/T189UWWVB71M9VRC.

30. Tornesello ML, Duraturo ML, Waddell KM, et al. Evaluating the role of human papillomaviruses in conjunctival neoplasia. *Br J Cancer*. 2006;94:446-449. doi:10.1038/sj.bjc.6602921.

31. Ateenyiagaba C, Weiderpass E, Tommasino M, et al. Papillomavirus infection in the conjunctiva of individuals with and without AIDS: An autopsy series from Uganda. *Cancer Lett*. 2006;239:98-102. doi:10.1016/j.canlet.2005.07.024.

32. Sen S, Sharma A, Panda A. Immunohistochemical localization of human papilloma virus in conjunctival neoplasias: A retrospective study. *Indian J Ophthalmol*. 2007;55:361. doi:10.4103/0301-4738.33822.

33. de Koning MN, Waddell K, Magyezi J, et al. Genital and cutaneous human papillomavirus (HPV) types in relation to conjunctival squamous cell neoplasia: A case-control study in Uganda. *Infect Agent Cancer*. 2008;3:12. doi:10.1186/1750-9378-3-12.

34. Guthoff R, Marx A, Stroebel P. No evidence for a pathogenic role of human papillomavirus infection in ocular surface squamous neoplasia in Germany. *Curr Eye Res*. 2009;34:666-671.

35. Manderwad GP, Kannabiran C, Honavar SG, et al. Lack of Association of High-Risk Human Papillomavirus in Ocular Surface Squamous Neoplasia in India. *Arch Pathol Lab Med*. 2009;133:5.

36. Simbiri KO, Murakami M, Feldman M, et al. Multiple oncogenic viruses identified in Ocular surface squamous neoplasia in HIV-1 patients. *Infect Agent Cancer*. 2010;5:6. doi:10.1186/1750-9378-5-6.

37. Ateenyi-Agaba C, Franceschi S, Wabwire-Mangen F, et al. Human papillomavirus infection and squamous cell carcinoma of the conjunctiva. *Br J Cancer*. 2010;102:262-267. doi:10.1038/sj.bjc.6605466.

38. Yu JJ, Fu P, Pink JJ, et al. HPV Infection and EGFR Activation/Alteration in HIV-Infected East African Patients with Conjunctival Carcinoma. Nicot C, ed. *PLoS ONE*. 2010;5:e10477. doi:10.1371/journal.pone.0010477.

39. Woods M, Chow S, Heng B, et al. Detecting Human Papillomavirus in Ocular Surface Diseases. *Investig Opthalmology Vis Sci*. 2013;54:8069. doi:10.1167/iovs.13-13140.

40. Carrilho C, Gouveia P, Yokohama H, et al. Human papillomaviruses in intraepithelial neoplasia and squamous cell carcinoma of the conjunctiva: a study from Mozambique. *Eur J Cancer Prev Off J Eur Cancer Prev Organ ECP*. 2013;22:566-568. doi:10.1097/CEJ.0b013e328363005d.

41. Afrogheh AH, Jakobiec FA, Hammon R, et al. Evaluation for High-risk HPV in Squamous Cell Carcinomas and Precursor Lesions Arising in the Conjunctiva and Lacrimal Sac: *Am J Surg Pathol*. 2016;40:519-528. doi:10.1097/PAS.0000000000000581.

42. Kamal S, Kaliki S, Mishra DK, et al. Ocular Surface Squamous Neoplasia in 200 Patients. *Ophthalmology*. 2015;122:1688-1694. doi:10.1016/j.ophtha.2015.04.027.

43. Baeten JM, McClelland RS, Richardson BA, et al. Vitamin A deficiency and the acute phase response among HIV-1-infected and -uninfected women in Kenya. *J Acquir Immune Defic Syndr 1999*. 2002;31:243-249.

44. Visser ME, Maartens G, Kossew G, et al. Plasma vitamin A and zinc levels in HIV-infected adults in Cape Town, South Africa. *Br J Nutr*. 2003;89:475. doi:10.1079/BJN2002806.

45. Shields CL, Shields JA. Tumors of the conjunctiva and cornea. *Surv Ophthalmol*. 2004;49:3-24. doi:10.1016/j.survophthal.2003.10.008.

46. Honavar S, Manjandavida F. Tumors of the ocular surface: A review. *Indian J Ophthalmol*. 2015;63:187. doi:10.4103/0301-4738.156912.

47. Erie JC, Campbell RJ, Liesegang TJ. Conjunctival and Corneal Intraepithelial and Invasive Neoplasia. *Ophthalmology*. 1986;93:176-183. doi:10.1016/S0161-6420(86)33764-3.

48. Gichuhi S, Macharia E, Kabiru J, et al. Clinical Presentation of Ocular Surface Squamous Neoplasia in Kenya. *JAMA Ophthalmol*. 2015;133:1305. doi:10.1001/jamaophthalmol.2015.3335.

49. Steffen J, Rice J, Lecuona K, et al. Identification of ocular surface squamous neoplasia by in vivo staining with methylene blue. *Br J Ophthalmol*. 2014;98:13-15. doi:10.1136/bjophthalmol-2013-303956.

50. Thomas BJ, Galor A, Nanji AA, et al. Ultra High-Resolution Anterior Segment Optical Coherence Tomography in the Diagnosis and Management of Ocular Surface Squamous Neoplasia. *Ocul Surf*. 2014;12:46-58. doi:10.1016/j.jtos.2013.11.001.

51. Nanji AA, Sayyad FE, Galor A, et al. High-Resolution Optical Coherence Tomography as an Adjunctive Tool in the Diagnosis of Corneal and Conjunctival Pathology. *Ocul Surf*. 2015;13:226-235. doi:10.1016/j.jtos.2015.02.001.

52. Atallah M, Joag M, Galor A, et al. Role of high resolution optical coherence tomography in diagnosing ocular surface squamous neoplasia with coexisting ocular surface diseases. *Ocul Surf*. 2017;15:688-695. doi:10.1016/j.jtos.2017.03.003.

53. Shousha MA, Karp CL, Canto AP, et al. Diagnosis of Ocular Surface Lesions Using Ultra–High-Resolution Optical Coherence Tomography. *Ophthalmology*. 2013;120:883-891. doi:10.1016/j.ophtha.2012.10.025.

54. Tole DM. Reliability of impression cytology for the diagnosis of ocular surface squamous neoplasia employing the Biopore membrane. *Br J Ophthalmol*. 2001;85:154-158. doi:10.1136/bjo.85.2.154.

55. Nolan GR, Hirst LW, Bancroft BJ. Efficacy of a training programme designed to teach cervical smear screeners to identify ocular surface squamous neoplasia using conjunctival impression cytology. *Cytopathology*. 1997;8:388-396. doi:10.1111/j.1365-2303.1997.tb00569.x.

56. Barros JN, Lowen MS, Ballalai PL, et al. Predictive index to differentiate invasive squamous cell carcinoma from preinvasive ocular surface lesions by impression cytology. *Br J Ophthalmol*. 2009;93:209-214. doi:10.1136/bjo.2008.147710.

57. Hoda RS. Non-gynecologic cytology on liquid-based preparations: A morphologic review of facts and artifacts. *Diagn Cytopathol*. 2007;35:621-634. doi:10.1002/dc.20698.

58. Rossi ED, Bizzarro T, Schmitt F, et al. The role of liquid-based cytology and ancillary techniques in pleural and pericardic effusions: An institutional experience: Pleural and Pericardic Effusions & ICC. *Cancer Cytopathol*. 2015;123:258-266. doi:10.1002/cncy.21518.

59. Martini M, Capodimonti S, Cenci T, et al. To Obtain More With Less: Cytologic Samples With Ancillary Molecular Techniques—The Useful Role of Liquid-Based Cytology. *Arch Pathol Lab Med*. 2018;142:299-307. doi:10.5858/arpa.2017-0148-RA.

60. Nanji AA, Mercado C, Galor A, et al. Updates in Ocular Surface Tumor Diagnostics: *Int Ophthalmol Clin*. 2017;57:47-62. doi:10.1097/IIO.0000000000000174.

61. Nanji AA, Sayyad FE, Karp CL. Topical chemotherapy for ocular surface squamous neoplasia: *Curr Opin Ophthalmol*. 2013;24:336-342. doi:10.1097/ICU.0b013e3283622a13.

62. Sayed-Ahmed IO, Palioura S, Galor A, et al. Diagnosis and medical management of ocular surface squamous neoplasia. *Expert Rev Ophthalmol*. 2017;12:11-19. doi:10.1080/17469899.2017.1263567.

63. Krilis M, Tsang H, Coroneo M. Treatment of Conjunctival and Corneal Epithelial Neoplasia with Retinoic Acid and Topical Interferon Alfa-2b: Long-term Follow-up. *Ophthalmology*. 2012;119:1969-1973. doi:10.1016/j.ophtha.2012.03.045.

64. Samarawickrama C, Chew S, Watson S. Retinoic acid and the ocular surface. *Surv Ophthalmol*. 2015;60:183-195. doi:10.1016/j.survophthal.2014.10.001.

65. Parrozzani R, Frizziero L, Trainiti S, et al. Topical 1% 5-fluoruracil as a sole treatment of corneoconjunctival ocular surface squamous neoplasia: long-term study. *Br J Ophthalmol*. 2017;101:1094-1099. doi:10.1136/bjophthalmol-2016-309219.

66. De Keizer RJW, De Wolff-Rouendaal D, Van Delft JL. Topical application of 5-Fluorouracil in premalignant lesions of cornea, conjunctiva and eyelid. *Doc Ophthalmol*. 1986;64:31-42. doi:10.1007/BF00166683.

67. Midena E, Angeli CD, Valenti M, et al. Treatment of conjunctival squamous cell carcinoma with topical 5-fluorouracil. *Br J Ophthalmol*. 2000;84:268-272. doi:10.1136/bjo.84.3.268.

68. Yeatts RP, Engelbrecht NE, Curry CD, et al. 5-Fluorouracil for the treatment of intraepithelial neoplasia of the conjunctiva and cornea. *Ophthalmology*. 2000;107:2190-2195. doi:10.1016/S0161-6420(00)00389-4.

69. Al-Barrag A, Al-Shaer M, Al-Matary N, et al. 5-Fluorouracil for the treatment of intraepithelial neoplasia and squamous cell carcinoma of the conjunctiva, and cornea. *Clin Ophthalmol Auckl NZ*. 2010;4:801-808.

70. Rudkin AK, Muecke JS. Adjuvant 5-fluorouracil in the treatment of localised ocular surface squamous neoplasia. *Br J Ophthalmol*. 2011;95:947-950. doi:10.1136/bjo.2010.186171.

71. Bahrami B, Greenwell T, Muecke JS. Long-term outcomes after adjunctive topical 5-flurouracil or mitomycin C for the treatment of surgically excised, localized ocular surface squamous neoplasia: Treatment of ocular surface neoplasia. *Clin Experiment Ophthalmol*. 2014;42:317-322. doi:10.1111/ceo.12184.

72. Joag MG, Sise A, Murillo JC, et al. Topical 5-Fluorouracil 1% as Primary Treatment for Ocular Surface Squamous Neoplasia. *Ophthalmology*. 2016;123:1442-1448. doi:10.1016/j.ophtha.2016.02.034.

73. Gichuhi S, Macharia E, Kabiru J, et al. Topical fluorouracil after surgery for ocular surface squamous neoplasia in Kenya: a randomised, double-blind, placebo-controlled trial. *Lancet Glob Health*. 2016;4:e378-e385. doi:10.1016/S2214-109X(16)30052-3.

74. Pe'er J. Ocular Surface Squamous Neoplasia: Evidence for Topical Chemotherapy. :13.

75. Kusumesh R, Ambastha A, Kumar S, et al. Retrospective Comparative Study of Topical Interferon a2b Versus Mitomycin C for Primary Ocular Surface Squamous Neoplasia. 2017;36:5.

76. Poole TRG. Conjunctival squamous cell carcinoma in Tanzania. *Br J Ophthalmol*. 1999;83:177-179. doi:10.1136/bjo.83.2.177.

77. Uganda Kaposi's Sarcoma Study Group, Newton R, Ziegler J, et al. The epidemiology of conjunctival squamous cell carcinoma in Uganda. *Br J Cancer*. 2002;87:301-308. doi:10.1038/sj.bjc.6600451.

78. Chisi S, Kollmann M, Karimurio J. Conjunctival squamous cell carcinoma in patients with Human Immunodeficiency Virus infection seen at two hospitals in Kenya. *East Afr Med J*. 2006;83. doi:10.4314/eamj.v83i5.9432.

79. Spitzer MS, Batumba NH, Chirambo T, et al. Ocular surface squamous neoplasia as the first apparent manifestation of HIV infection in Malawi. *Clin Experiment Ophthalmol*. June 2008. doi:10.1111/j.1442-9071.2008.01794.x.

80. Ogun GO, Ogun OA, Bekibele CO, et al. Intraepithelial and invasive squamous neoplasms of the conjunctiva in Ibadan, Nigeria: a clinicopathological study of 46 cases. *Int Ophthalmol*. 2009;29:401-409. doi:10.1007/s10792-008-9257-8.

81. Furahini G, Lewallen S. Epidemiology and Management of Ocular Surface Squamous Neoplasia in Tanzania. *Ophthalmic Epidemiol*. 2010;17:171-176. doi:10.3109/09286581003731544.

82. Makupa II, Swai B, Makupa WU, et al. Clinical factors associated with malignancy and HIV status in patients with ocular surface squamous neoplasia at Kilimanjaro Christian Medical Centre, Tanzania. *Br J Ophthalmol*. 2012;96:482-484. doi:10.1136/bjophthalmol-2011-300485.

83. Tiong T, Borooah S, Msosa J, et al. Clinicopathological review of ocular surface squamous neoplasia in Malawi. *Br J Ophthalmol*. 2013;97:961-964. doi:10.1136/bjophthalmol-2012-302533.

84. Osahon AI, Ukponmwan CU, Uhunmwangho OM. Prevalence of HIV seropositivity among patients with squamous cell carcinoma of the conjunctiva. *Asian Pac J Trop Biomed*. 2011;1:150-153. doi:10.1016/S2221-1691(11)60015-4.

85. Starita N, Annunziata C, Waddell KM, et al. Identification of Human Herpesvirus 8 Sequences in Conjunctiva Intraepithelial Neoplasia and Squamous Cell Carcinoma of Ugandan Patients. *BioMed Res Int*. 2015;2015. doi:10.1155/2015/801353.

86. Nguena MB, van den Tweel JG, Makupa W, et al. Diagnosing Ocular Surface Squamous Neoplasia in East Africa. *Ophthalmology*. 2014;121:484-491. doi:10.1016/j.ophtha.2013.09.027.

87. Gichuhi S, Macharia E, Kabiru J, et al. Toluidine Blue 0.05% Vital Staining for the Diagnosis of Ocular Surface Squamous Neoplasia in Kenya. *JAMA Ophthalmol*. 2015;133:1314. doi:10.1001/jamaophthalmol.2015.3345.

88. Lloyd H-WCM, Arunga S, Twinamasiko A, et al. Predictors of Ocular Surface Squamous Neoplasia and Conjunctival Squamous Cell Carcinoma among Ugandan Patients: A Hospital-based Study. *Middle East Afr J Ophthalmol*. 2018;25:150-155. doi:10.4103/meajo.MEAJO_187_16.

89. Lecuona K, Stannard C, Hart G, et al. The treatment of carcinoma in situ and squamous cell carcinoma of the conjunctiva with fractionated strontium-90 radiation in a population with a high prevalence of HIV. *Br J Ophthalmol*. 2015;99:1158-1161. doi:10.1136/bjophthalmol-2014-306327.

90. Waddell KM, Lewallen S, Lucas SB, et al. Carcinoma of the conjunctiva and HIV infection in Uganda and Malawi. *Br J Ophthalmol*. 1996;80:503-508. doi:10.1136/bjo.80.6.503.

91. Waddell KM, Downing RG, Lucas SB, et al. Corneo-conjunctival carcinoma in Uganda. *Eye*. 2006;20:893-899. doi:10.1038/sj.eye.6702043.

92. Gichuhi S, Kabiru J, M'bongo Zindamoyen A, et al. Delay along the care-seeking journey of patients with ocular surface squamous neoplasia in Kenya. *BMC Health Serv Res*. 2017;17. doi:10.1186/s12913-017-2428-4.

93. Waddell K, Kwehangana J, Johnston WT, et al. A case-control study of ocular surface squamous neoplasia (OSSN) in Uganda. *Int J Cancer*. 2010:NA-NA. doi:10.1002/ijc.25040.

94. Katsekera E, Tanyanyiwa L, Chikwasha V, et al. Screening for Ocular Surface Squamous Neoplasia (OSSN) by Slit-lamp Assisted Visual Inspection (SAVI) following a short course of mild topical steroid-test qualities. *Afr Vis Eye Health*. 2014;73. doi:10.4102/aveh.v73i1.3.

11. Appendix A: Studies with primary or adjuvant use of 5FU for OSSN

Author (year)	Sample characteristics (age, sex, HIV)	Diagnosis (CIN vs invasice squamous neoplasia)	Primary vs adjuvant	Regimen and cycles	Recurrence	Side effects	Comments
Keizer[66] (1986)	n = 4<br>M:F, 3:1<br>Mean age: 63 years	CIN I-III<br>CIN_{IS}<br>SCC	Primary and adjuvant	1% 5FU given 3-5 times per day<br><br>No fixed regimen was used	n = 1	None	
Midena[67] (2000)	n = 8<br>M:F, 3:1<br>Mean age: 70 years<br>Mean follow-up: 27 months	Undefined OSSN	Primary n = 2<br>Adjuvant n = 6	1 cycle = 5FU 1% QID for 4 weeks<br><br>All patients had 1 cycle	n = 1 (at 6 months)	n = 8<br>Conjunctivitis and superficial keratitis	Recurrence resolved with 1 cycle of 5FU.<br><br>HIV unknown
Yeats[68] (2000)	n = 7<br>All male<br>Mean age: 74 years<br>Mean follow-up: 18.5 months	CIN I-III<br>CIN_{IS}	Primary n = 6<br>Adjuvant n = 1	1 cycle = 5FU 1% QID for 2-4 days<br><br>Cycle repeated every 30-40 days<br><br>Mean of 3.75 cycles used	n = 3	None	1 5FU treatment failure that was resolved with MMC
Al-Barrag[69] (2010)	n = 15<br>M:F, 1:4<br>Mean age: 51 years<br>Median follow-up: 15 months	CIN I-III<br>CIN_{IS}<br>SCC	Adjuvant to surgery	1 cycle = 1% 5FU given QID for 4 days with 30 days drug holiday<br><br>All patients received 6 cycles of treatment	n = 1, this patient received an additional 6 cycles of 5FU	No mentioned in the report	All patients received a subconjunctival dose of 5FU, 5mg, at the end of surgery

Rudkin[70] (2011)	n = 65<br>M:F, 3:1<br>Mean age: 66 years<br>Mean follow-up: 23 months	CIN I-III	Adjuvant to surgical excision with 2mm margins and double cryo	1% 5FU given QID for 2 weeks only	n = 1, but this patient did not complete the 2 week course of 5FU	n = 37<br>Mostly mild, but some severe lid toxicity.<br>n = 4, stopped 5FU due to the side effects	Lid toxicity was a concern in this study with transient ectropion developing in 1 patient
Bahrami[71] (2014)	n = 89<br>M:F, 3:1<br>Mean age: 67 years<br>Median follow-up: 34 months	CIN I-III	Adjuvant to surgical excision with 2mm margins and double cryo	1% 5FU given QID for 2 weeks only	n = 1, but this patient did not complete the 2 week course of 5FU	n = 61, all where transient in nature.	Transient lid toxicity was the main side effect.
Joag[72] (2016)	n = 44<br>M:F, 2.7:1<br>Mean age: 68 years<br>Mean follow-up:	CIN I-III<br>CIN_{IS}<br>SCC	Primary n = 44	1 cycle = 5FU 1% QID for 1 week with 3-week drug holiday<br><br>Mean of 4 cycles used	n = 4 of the 36 who completed 5FU<br><br>Nodular morphology increased by 11-fold	n = 27 temporary mild side effects<br><br>n = 1, discontinued therapy due to side effects	4 non-responders<br>4 treatment failures (switched to another agent)
Gichuhi[73] (2016)	n = 98 (49 in the 5FU treatment and 49 in the control arm)<br>M:F, 1:2<br>Mean age: 41 years	CIN I-III<br>CIN_{IS}<br>SCC	Adjuvant after surgical excision with 4mm margins and **NO** cryotherapy	1% 5FU QID given post-op for 4 weeks only	Treatment arm: 11% recurrence<br><br>Control arm: 36% recurrence	Transient side effects noted	The surgery involved 4mm margins and **NO** cryotherapy was used. The adjuvant use of 5FU reduced the recurrence rate.
Parrozzani[65] (2017)	n = 41<br>M:F, 1.7:1<br>Mean age: 68 years<br>Mean follow-up: 105 months	CIN I-III<br>CIN_{IS}<br>SCC	Primary: n = 41	1 cycle = 5FU 1% QID for 4weeks, with 4 weeks drug holiday<br><br>Mean of 1.5 cycles used	n = 4	n = 19 temporary mild side effects	7 incomplete responders<br>Response to therapy was associated with a tumour thickness of <1.5mm

12. Appendix B: Studies of OSSN in the African context

Author (year)	Sample	Objectives	Key Findings
**Epidemiology**			
Poole[76] (1999)<br><br>Tanzania	n = 211<br>Mean age: 45 years<br>M:F. 1:1	To assess the change in incidence of OSSN over a 22 year period (1976 - 1997)	• There was a large increase in cases in the last three years of the study<br>• The mean age remained consistent over the study period
Newton[77] (2002)<br><br>Uganda	n = 1274 (60 cases and 1214 control)<br><br>M:F, 1:1	To describe the epidemiology of OSSN in Uganda.	• OSSN was associated with HIV (OR, 10)<br>• Seroprevalence of HPV 18, 45 was too low to comment<br>• HPV-16 anti-bodies were not significantly associated with OSSN
Waddell[91] (2006)<br><br>Uganda	n = 476<br>Mean age: 32 years<br>M:F, 1:1.2	To describe a large series of OSSN cases in Uganda that received uniform surgical management.	• Strong association between OSSN and HIV (64% of OSN patients were HIV positive)<br>• Surgery used a 3mm margin but did not use cryotherapy<br>• Recurrence rate was 3.2% with a mean follow-up of 32 months<br>• 50% of the recurrences were from CIN lesions and all were in HIV positive patients<br>• Most tumours were interpalpebral
Chisi[78] (2006)<br><br>Kenya	n = 409<br><br>Mean age: 38 years<br><br>M:F, 1:1	Cross-sectional study to determine the prevalence of OSSN in a HIV positive cohort at two hospitals in Kenya.	• Prevalence of OSSN was 8%<br>• There was no sex predilection<br>• Patients presented late with advanced disease<br>• 31% were recurrent lesions<br>• Presenting complaints were non-specific
Spitzer[79] (2008)<br><br>Malawi	n = 38<br><br>Mean age: 33 years<br><br>M:F, 1.4:1	To determine the percentage of patients where OSSN is the primary manifestation of HIV.	• None had had a previous HIV test<br>• 70% were HIV positive<br>• There were no other clinical features of HIV<br>• OSSN can be the first manifestation of HIV infections

Ogun[80] (2009)<br><br>Nigeria	n = 46<br>Mean age: 53 years<br>M:F, 1:1	To review the clinic-pathological features of OSSN.	• 60% of patients presented with orbital invasion<br>• 10% of patients had lymph node metastasis (all T3 or higher)<br>• Average time to presentation was 2 years<br>• 46% had koilocytic changes which is associated with HPV infection
Furahini[81] (2010)<br><br>Tanzania	Referral hospitals, n = 921<br>M:F, 1:1.6<br><br>Regional centres, n = 729<br>M:F, 1:1.7	To estimate the incidence of OSSN by region in Tanzania.	• Annual incidence was 2.2 per 100 000 persons<br>• Regional incidence rates correlated significantly with regional HIV infection rates<br>• These lesions were not histologically confirmed, only clinically suspected
Makupa[82] (2012)<br><br>Tanzania	n = 150 (132 OSSN, 18 benign)<br>Mean age: 39 years<br>M:F, 1:2	To describe the clinical characteristics of OSSN in SSA according to HIV status.	• 60% of OSSN cases were HIV positive<br>• Mean CD4 count was 71 cells/µl<br>• Lesions in the HIV cohort were more likely to be a higher-grade malignancy
Tiong[83] (2013)<br><br>Malawi	n = 58<br>Mean age: 36 years<br>M:F, 1:3	To identify clinical factors associated with the histopathological diagnosis of OSSN.	• Three factors were found to be associated with invasive SCC:<br>• Tumour size greater than 20.5mm^2<br>• HIV seropositivity<br>• Male gender
Steele[3] (2015)<br><br>Botswana	n = 468<br>Mean age: 38 years<br>M:F, 1:1	To describe the presentation and management of patients with OSSN at a tertiary centre in Botswana.	• 39% were HIV infected<br>• 47% had a CD4 <200<br>• 7% recurrence rate (treatment was with excision or excision with strontium 90 adjuvant)<br>• Median time to recurrence, 6 months
Gichuhi[92] (2017)<br><br>Kenya	n = 158<br>M:F, 1:2<br>Mean age: 42 years	To describe the presentation and referral journey for patients with OSSN.	• Females presented late than males<br>• Indirect referral delayed surgery from 5.5 to 9.6 months<br>• The delay did not affect the mean tumour size

Risk Factors			
Waddell[90] (1996)<br><br>Uganda	n = 53 (38 OSSN, 15 controls)<br>Mean age: 35 years<br>M:F, 1:1.7	To describe the association between HIV and HPV in OSSN and benign lesions.	• 71% of OSSN patients were HIV positive vs 16% of controls<br>• 35% were HPV16 positive in the OSSN group vs 13% in the controls
Ateenyi-Agaba[12] (2004)<br><br>Uganda	n = 43 (21 cases, 22 controls)	To assess the presence of TP53 mutations in biopsies of OSSN and controls in an area with high UV-exposure and HIV prevalence.	• TP53 mutations were found in 52% of cases and 14% of controls.<br>• The controls were pingueculae and pterygia<br>• 50% of all the mutations were CC → TT transitions, characteristic of UV-induced mutagenesis
Tornesello[15] (2005)<br><br>Uganda	n = 222 (107 OSSN, 115 controls)	To analyse the distribution and role of TP53 polymorphisms in OSSN.	• TP52 Arg/Arg codon 72 genotype is a risk for invasive SCC and CIN3 lesions of the conjunctiva in Uganda
Waddell[93] (2010)	n = 1080 (318 OSSN, 762 controls)	To identify risk factors for OSSN other than HIV infection.	• Increasing reported sun exposure was associated with an increasing OR of OSSN<br>• Previous ocular injury was a risk for OSSN<br>• Pingueculae was a risk for OSSN
Osahon[84] (2011)<br><br>Nigeria	n = 33<br>Mean age: 39 years<br>M:F, 1:1.5	To determine the association between OSSN and HIV.	• 75% of patients with OSSN had HIV<br>• Decreasing mean age compared to earlier African studies
Starita[85] (2015)<br><br>Uganda	n = 72	To analyse the prevalence of HHV8 in OSSN.	• HHV8 was found in 19% of OSSN lesions and 7% of controls<br>• There was no relationship between the rate of infection and the stage of OSSN
Gichuhi[10] (2016)<br><br>Kenya	n = 262 (131 cases, 131 controls)<br>Mean age: 42 years<br>M:F, 1:2	To identify modifiable risk factors for the development of OSSN.	• HIV increased risk<br>• Allergic conjunctivitis increased risk<br>• Smoking was not found to affect risk<br>• Sun exposure was a risk factor

**Diagnosis**			
Nguena[86] (2014)<br><br>Tanzania	n = 120 (60 OSSN and 60 controls)<br>Mean age: 44 years<br>M:F, 1:1	To assess the diagnostic accuracy of clinical examination to distinguish OSSN from benign lesions. To evaluate the utility of confocal microscopy in distinguishing OSSN from benign lesions.	• Clinical signs found more in the OSSN group: feeder vessels, leukoplakia, gelatinous appearance<br>• There was a strong association between HIV and OSSN (OR, 24)<br>• Correct clinical diagnosis ranged from 56 to 86%<br>• Confocal was not reliably able to distinguish OSSN from benign lesions (sensitivity: 39%, specificity: 67%)
Katsekera[94] (2014)<br><br>Zimbabwe	n = 119<br>Mean age: 42 years<br>M:F, 1:2	To evaluate the accuracy of a slit-lamp assisted visual inspection screening tool for the diagnosis of OSSN.	• The vascularity, granularity and conjunctival lustre was assessed after a week of topical FML 0.1%<br>• Sensitivity = 94%<br>• Specificity = 74%<br>• PPV = 75%<br>• NPV = 94%
Steffen[49] (2014)<br><br>South Africa	n = 75<br>Median age: 35 years<br>M:F, 1:1.5	To determine the diagnostic accuracy of 1% methylene blue stain to diagnose OSSN.	• Sensitivity of 97%<br>• Specificity 50%<br>• Positive predictive value 60%<br>• Negative predicative value 96%
Gichuhi[87] (2015)<br><br>Kenya	n = 419<br>Mean age: 37 years<br>M:F, 1:2	To evaluate the safety and accuracy of Toluidine blue 0.05% in distinguishing histologically confirmed OSSN from other conjunctival lesions.	• No corneal toxicity was found<br>• Toluidine blue had a sensitivity of 92%, specificity 31%, PPV 41% and NPV 88% for OSSN diagnosis<br>• It is therefore useful to exclude but not confirm OSSN
Gichuhi[48] (2015)<br><br>Kenya	n = 496<br><br>Mean age: 41 years<br><br>M:F, 1:2	To describe the clinical appearance of OSSN to help distinguish them from benign conjunctival lesions.	• OSSN were more likely to be at the temporal limbus, circumlimbal, be inflamed and have leukoplakia<br>• Using clinical features to diagnose OSSN had a sensitivity of 85%, specificity of 60% and positive predictive value of 54%<br>• Exposure to cigarette smoking was found to be a risk factor

Lloyd[88] (2018)<br><br>Uganda	n = 212<br><br>Mean age: 48 years<br><br>M:F, 1:1.2	To assess the clinical features that distinguish OSSN from benign lesions.	• Clinical features of OSSN: rough tumour surface, interpalpebral location, six or more feeder vessels<br>• No association with sun exposure<br>• Higher rate of HIV than the benign cohort
**Management**			
Lecuona[89] (2015)<br><br>South Africa	n = 69<br><br>Mean age: 42 years<br><br>M:F, 1:1.3	To describe the sole adjuvant use of Strontium-90 brachytherapy for OSSN. Two plaque sizes where used, 8.5mm (n=49) and 12mm (n=20).	• Recurrence rate of 11.6% (all with the smaller 8.5mm plaque)<br>• Side effects included dry eye in five patients and 1D of astigmatism in another<br>• 47% of the cohort was HIV positive
Gichuhi[73] (2016)<br><br>Kenya	n = 98 (49 in the 5FU treatment and 49 in the control arm)<br>M:F, 1:2<br>Mean age: 41 years	To assess whether adjuvant 1% 5FU (QID for 4 weeks) given after surgery without cryotherapy could reduce the recurrence rates of OSSN.	• Recurrence rate in the treatment arm was 11%<br>• Recurrence rate in the control arm was 36%<br>• Side effects were mild and transient
**Human Papilloma Virus**			
Moubayed[29] (2004)	n = 14	To describe the presence of HPV 6, 11, 16 and 18 in histologically proven OSSN specimens, with two PCR techniques.	• Conventional in situ hybridisation showed HPV in 5 of 14 specimens<br>• ImmunoMax PCR showed HPV in 13 of 14 specimens<br>• They did not test for cutaneous HPV
Tornesello[30] (2006)<br><br>Uganda	n = 149 (86 OSSN and 63 controls)<br><br>Median age OSSN: 32 years<br><br>Median age controls: 30 years	To determine the prevalence of mucosal and cutaneous HPV in OSSN and conjunctiva of HIV-positive and HIV-negative patients.	• HPV positive in 20% of cases<br>• HPV positive in 2% of controls<br>• HPV positivity not related to stage of OSSN<br>• No pattern of preference seen between mucosal or cutaneous HPV types

Ateenyi-Agaba[31] (2006)<br><br>Uganda	AIDS group, n = 33<br>Mean age: 30 years<br>M:F, 1:1<br><br>Infectious diseases group, n = 45<br>Mean age: 40 years<br>M:F, 1.8:1<br><br>Chronic ds/trauma group, n = 58<br>Mean age: 44<br>M:F, 2.6:1	To compare the HPV prevalence in conjunctival biopsies between patients that died from AIDS to patients who died from other causes.	• Only cutaneous HPV was detected in the biopsies from all groups<br>• The prevalence of cutaneous HPV did not differ between the group with AIDS and group that died of other causes<br>• This implies that HIV does not enhance the rate of cutaneous HPV infection<br>• Therefore, if there is a higher infection rate of HPV in OSSN, this must be causative to the development of OSSN
De Koning[33] (2008)<br><br>Uganda	OSSN, n = 81<br>Mean age: 35 years<br><br>Controls, n = 29<br>Mean age: 30 years	An investigation of the role of genital and cutaneous HPV in the aetiology of OSSN.	• Prevalence of genital HPV was higher than cutaneous HPV in both groups<br>• Prevalence of genital HPV was not different between the groups<br>• Prevalence of cutaneous HPV significantly higher in the OSSN vs control group<br>• Cutaneous HPV prevalence did not change with histological grade of OSSN
Simbiri[36] (2010)<br><br>Botswana	n = 39<br>M:F, 1:2	To identify viral etiologic agents in OSSN in HIV infected patients in Botswana.	• EBV in 83%<br>• HPV in 75%, they only tested to mucosal HPV types with 11, 16, 18 the most common<br>• Kaposi sarcoma herpes virus in 70%<br>• CMV in 61%<br>• HSV in 70%<br>• There was a high degree of co-infection

Ateenyi-Agaba[37] (2010)<br><br>Uganda	SCCC, n = 94<br>Mean age: 37 years<br><br>CIN, n = 39<br>Mean age: 33 years<br><br>SCCC/CIN, M:F, 1:1.3<br><br>Controls, n=285<br>Mean age: 34 years<br>M:F, 1:1.1	Case-control study in SCCC and dysplasia to assess the role of HIV and HPV and risk factors.	• Cutaneous HPV was significantly associated with SCCC and dysplasia<br>• Mucosal HPV was NOT associated with SCCC or dysplasia<br>• HPV 5, 8, 14, 17, 23 were the most common types identified<br>• HPV 8 incidence increase as lesions worsen<br>• Cutaneous HPV in SCCC/dysplasia was mainly seen in the HIV patients<br>• Half the SCCC/dysplasia group had no HPV infection
Yu[38] (2010)<br><br>Uganda	n = 38	To assess the relationship between HPV infection and EWGRF signalling in OSSN.	• 61% HPV 18 positive<br>• Possible mechanism identified whereby oncoproteins E5, E6, E7 activate the RGRF cascade and lead to unchecked cellular proliferation
Carrilho[40] (2013)<br><br>Mozambique	SCCC/CIN, n = 19<br><br>Controls, n = 3<br><br>Median age: 35 years	Case-control study to assess the relationship between HPV and SCCC/dysplasia.	• 58% HPV positive in the SCCC/dysplasia cohort<br>• Predominant HPV were the cutaneous types<br>• No HPV detected in the controls

13. Appendix C: Information Sheet and Consent Form

14. Appendix D: Study Participant Details

Study Number	Name	Surname	Medical Record Number	Contact Number 1	Contact Number 2
1					
2					
3					

15. Appendix E: Data Collection Sheet

16. Appendix F: Data Analysis

Objective	Study Design	Variables	Measurement Scale	Analysis
1. Describe the characteristics of patients with OSSN as St John Eye Hospital	Observational	- Demographics<br>- Age*<br>- Gender<br>- Race<br>- Symptoms on presentation<br>- Pain<br>- Foreign body sensation<br>- Epiphora<br>- Itching<br>- Reduced vision<br>- Entry into the health system<br>- Local clinic<br>- Regional ophthalmic unit<br>- Tertiary ophthalmic unit<br>- Private ophthalmologist<br>- Optometrist<br>- General practitioner<br><br>- Clinical features<br>- Visual acuity<br>- Clinical features of mass<br>- Location and size<br>- Morphology<br>- HIV<br>- Status<br>- CD4*<br>- Viral load*<br>- Anti-retroviral therapy use<br>- Use of systemic immunosuppression<br>- Tobacco use<br>- Cigarette smoking	Categorical	Categorical variables: Number, percentage and 95% confidence interval<br><br>Continuous variables normally distributed: mean, standard deviation and 95% confidence interval<br><br>Continuous variables not normally distributed and ordinal variables: median and interquartile range

		- Snuff<br>- Chewing tobacco<br>- History of ocular injury<br>- Penetrating<br>- Blunt<br>- Chemical<br>- Thermal<br>- UV exposure (expressed as average number of hours spent in the sun in an average day)<br>- Chronic ocular inflammatory disease<br>- Chronic allergic conjunctivitis<br>- Steven Johnson Syndrome<br>- Mucous membrane pemphigoid<br>- Petroleum chemical exposure<br>- HBV and HCV infections<br>- Serum vitamin A levels*		
2. Compare the non-invasive diagnostic methods with histology	Case-control	- Histology<br>- CIN<br>- Carcinoma in-situ<br>- Invasive squamous cell<br>- Benign<br>- Mass margin involvement<br>- Impression cytology<br>- Negative<br>- Atypical<br>- Suspicious<br>- Malignant<br>- AS-OCT<br>- Epithelial thickness*<br>- Transition zone<br>- Clear plane<br>- Methylene blue stain	Categorical<br><br>*Continuous	Chi square<br><br>*ROC curve
2. Outcomes of OSSN management	Observational	- Treatment success<br>- Surgery<br>- 5FU	Categorical	number, percentage, 95% confidence interval, Kaplan-meier curve

		- 5FU + retinoic acid		
		- Recurrence rates<br>- Surgery<br>- 5FU<br>- 5FU + retinoic acid	Continuous	number, percentage, 95% confidence interval, Kaplan-meier curve
3. Factors associated with success and recurrence	Observational	- Tumour size*<br>- Tumour thickness<br>- Tumour morphology<br>- CD4 at diagnosis of HIV*<br>- CD4 at diagnosis of OSSN*<br>- VL at diagnosis of HIV*<br>- VL at diagnosis of OSSN*<br>- ARV use	Categorical<br><br>*Continuous	COX proportional hazard ratio
4. Adverse events associated with management	Observational	- Surgery related complications<br>- Toxicity of topical 5FU and retinoic acid	Categorical	Number, percentage and 95% confidence interval

17. Appendix G: Budget

Item	Use	Number Needed	Cost per unit	Total
**Impression cytology filter papers for collection**	Collection of impression cytology specimen	650	R14	R9 100<br><br>(Paid from RINC fund)
**Methylene Blue**	Vital dye for diagnosis	1	R250	R250<br><br>(Paid from RINC fund)
**Retinoic Acid**	Adjunct to 5FU	75	-	Provided by compounding pharmacy
**5FU**	Topical chemotherapy drop	100	-	Standard treatment provided by hospital pharmacy
**Cytology laboratory filters**	For the processing of the impression cytology specimen	325	R70	R22 750
			**Total**	**R32 100**

**Funding Source**	**Value**
Carnegie Research Fund	R120 000
RINC Fund	R7 000
